# A state of stochastic cancer stemness through the CDK1-SOX2 axis

**DOI:** 10.18632/oncotarget.26819

**Published:** 2019-04-05

**Authors:** Dinoop Ravindran Menon, Mayumi Fujita

**Affiliations:** ^1^ Department of Dermatology, University of Colorado Denver, Aurora, CO, USA; ^2^ Denver VA Medical Center, Denver, CO, USA; ^3^ Department of Immunology and Microbiology, University of Colorado Denver, Aurora, CO, USA

**Keywords:** cancer stemness, melanoma, immune evasion, CDK1, SOX2

## Abstract

The concept of cancer stemness has undergone a paradigm shift during the last decade where there is wider acceptance of the idea that stemness in cancer is a more dynamic and plastic phenomenon than previously thought. However, we have yet to understand the mechanisms on how this stochastic plasticity arises and is maintained. Recently, we have shown that CDK1 plays a critical role in stochastic stemness and tumor initiation potential through regulating SOX2 phosphorylation in multiple cancer types. The phosphorylation of SOX2 affects its nuclear localization, thereby determining the transcriptional fate of its downstream targets. We have also validated the significance of these findings using clinical samples by demonstrating that CDK1^high^ tumor samples displayed upregulation of MYC target genes, which were reported to overlap with SOX2 targets. In the current article, we further discuss the possibility of a closed, feed-forward loop between SOX2 and CDK1 through a long non-coding RNA, CCAT1, which would explain the sustained activation of this loop. Despite the extensive investigation of the cancer stemness as a cause of drug resistance, its role in immune evasion still requires further understanding, and hence, in this article, we further discuss the possibility of this CDK1-SOX2 axis contributing to immune resistance through modulating cell-to-cell interaction directly or indirectly in the tumor microenvironment.

Cancer stemness is a concept that has evolved with our understanding of the nature of cancer cells. In certain cancer types such as colon cancer and squamous cell carcinoma, lineage-tracing studies using mouse models have pointed to a hierarchical model of cancer development where epithelial stem cells have been identified as the cause of tumor initiation. However, cancer cells exhibit an exorbitant plasticity in their phenotypes after the transition into a cancerous state, and even adult stem cells can exist in a state of constant flux or non-hierarchical plasticity in the tissue microenvironment, all of which implicate a shift from a hierarchical rigid model of cancer stem cells to a non-hierarchical or phenotypically plastic model [1].

Despite numerous studies investigating the phenotypic markers and functional consequences of the plastic state of cancer cells, there is a dearth of information available on the molecular mechanisms regulating this plasticity. Our recent study sheds light on this field by elucidating a novel mechanism of CDK1 contributing to cancer stemness. We have demonstrated an upregulation of CDK1 in a subpopulation of melanoma and colon cancer cells with high MHC I expression that displayed high tumor initiating potential [2]. The expression of MHC I in these cells was driven by CDK1 through the NF-kB pathway and resulted in phenotypic plasticity, indicating a non-hierarchical stochastic switching between stem-like and non-stem-like states. It needs to be noted that tumor initiating potential is different from tumor growth rate: the former represents an ability of cancer cells to initiate or form tumors whereas the latter is associated with the speed of tumor growth. Portrayed by this difference, modulating CDK1 activity altered tumor initiating potential without affecting cell proliferation in 2D assays. These findings illustrate a CDK1-mediated plasticity and stem-like phenotype in cancer cells.

We have also delineated an indispensable role for the stem cell transcription factor SOX2 in CDK1-mediated stemness and tumorigenesis by demonstrating the interaction of CDK1 with SOX2 in cancer cells. SOX2 is known to be instrumental in tumorigenesis in multiple cancer types [3, 4]; however, its role in melanoma has been controversial [2, 5-7]. Whereas some reports, including ours, revealed the importance of SOX2 in tumor initiation [2] [5], others demonstrated an opposite trend using mouse melanoma models [6, 7]. Functional consequences of SOX2 depend on its expression, post-translational modification, subcellular localization and interaction with other transcription factors, which might explain the discrepancy in the role of SOX2 reported in various studies. By dissecting transcriptional activities and nuclear localization of SOX2, we have uncovered a vital role of CDK1 in governing the transcriptional activity of SOX2 through its phosphorylation at S249-S250-S251, the sites required for nuclear translocation of SOX2 [2].

Furthermore, we have validated the clinical significance of our findings by unveiling the involvement of CDK1 in MYC target genes, mTOR signaling and DNA repair pathways in patient tumors from melanoma, colon and pancreatic cancer. Among these signatures, the upregulation of MYC target genes is of particular importance because MYC can bind to up to 85% of the same promoters as SOX2, amplifying its downstream targets including various CDKs [8]. A previous report has shown that CDK1 is indispensable for survival of cancer cells overexpressing MYC [9]. Therefore, we speculated crosstalk between CDK1 and SOX2/MYC in melanoma, and silenced SOX2 in human melanoma cells. Consistent with our hypothesis, we found downregulation of CDK1 after SOX2 knockdown in these cells (Figure 1A and 1B). Recent reports have demonstrated that both SOX2 [10] and MYC [11] activate colon cancer-associated transcript 1 (CCAT1), a long non-coding RNA. CCAT1 upregulates CDK1 in liver cancer cells by binding to and sponging miR-490-3p, a micro RNA known to downregulate CDK1 expression [12]. Together with our previous study showing the regulation of SOX2 by CDK1, these data indicate the presence of a closed, feed-forward loop in the CDK1-SOX2 axis in melanoma, as depicted in Figure 1C.

**Figure 1:**
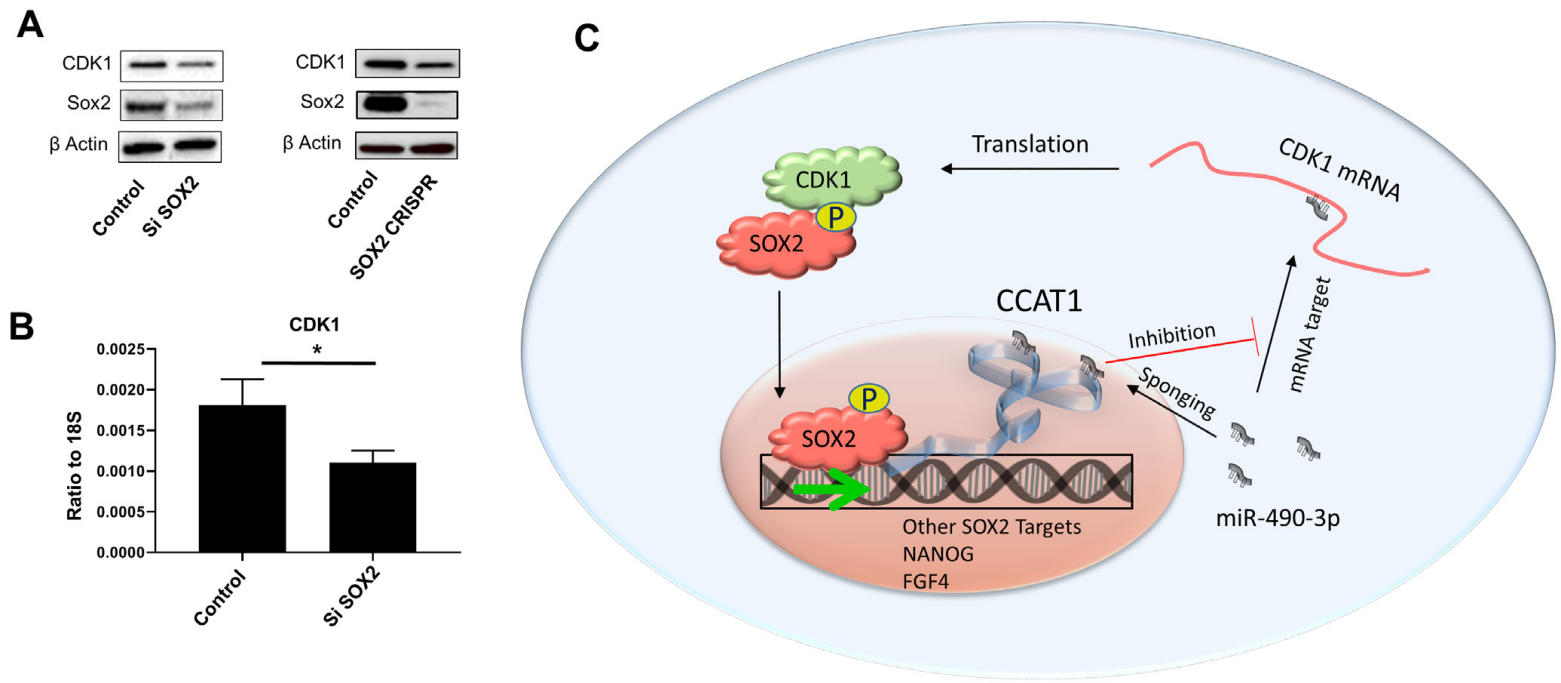
SOX2 regulates CDK1 through a feed-forward loop **A.** Sox2 knockdown by siRNA (left) or CRISPR (right) in A375 cells downregulates CDK1 protein expression. **B.** SOX2 siRNA knockdown in A375 cells downregulates CDK1 mRNA expression. **C.** Graphical representation of a possible feed-forward loop regulating CDK1 and SOX2.

Another emerging field of interest is the role of cancer stemness in immune evasion. Immunotherapies have shown to be effective in many cancer types including melanoma; however, only a minority of patients exhibit a durable and sustained response to immunotherapies [13]. Accumulating evidence suggests an important role for SOX2 in cancer immune evasion [14, 15]. Fox example, Dickkopf-related protein 1 (DKK1), induced by SOX2, positively regulates myeloid-derived suppressor cells in the tumor microenvironment [14] and provides metastatic latency and immune evasion in lung cancer by protecting cells from NK cells [15]. NANOG, an embryonic stem cell-related gene and another target of SOX2 [2], is also shown to induce resistance to lysis by cytotoxic T cells in cervical cancer [16].

Altogether, our study provides a mechanistic perspective on cancer progression through CDK1-SOX2-mediated cancer stemness and immune evasion of tumor cells. Hence, targeting the CDK1-SOX2 axis would hold immense promise in future cancer therapeutics, and warrants further investigation.
